# Serum 25 (OH) D levels and risk of female-specific cancer in premenopausal women: a prospective study

**DOI:** 10.3389/fnut.2025.1617565

**Published:** 2025-09-15

**Authors:** Yue Hu, Chen Zhu, Yingyi Qin, Ying Zhu, Jianzheng Zhang, Qiang Tong, Shengyun Cai

**Affiliations:** ^1^Department of Obstetrics and Gynecology, The First Affiliated Hospital of Naval Medical University, Shanghai, China; ^2^College of Economics and Management, China Agricultural University, Beijing, China; ^3^Department of Military Health Statistics, Naval Medical University, Shanghai, China; ^4^Department of Pulmonary and Critical Care Medicine, 7th Medical Center of Chinese PLA General Hospital, Beijing, China; ^5^College of Pulmonary and Critical Care Medicine, 8th Medical Center of Chinese PLA General Hospital, Beijing, China; ^6^Department of Orthopedics, The Forth Medical Center, PLA General Hospital, Beijing, China; ^7^Department of Rheumatology and Immunology, Shanghai Sixth People's Hospital Affiliated to Shanghai Jiao Tong University School of Medicine, Shanghai, China; ^8^Division of Rheumatology and Clinical Immunology, Department of Medicine, Queen Mary Hospital, University of Hong Kong, Hong Kong, Hong Kong SAR, China

**Keywords:** female-specific malignant tumor, premenopausal women, prospective cohort study, serum 25 (OH) D, UK Biobank, vitamin D

## Abstract

**Background:**

Serum 25 (OH) D levels are associated with various diseases, including cancers, but inconsistencies exist for female-specific malignancies. This study is aimed to explore the real relationship between serum 25 (OH) D levels and incidence rates of female specific cancers in premenopausal women by a large-scale prospective cohort study.

**Study design:**

We analyzed data from 51,286 UK Biobank participants using Cox regression models to explore associations. Subgroup analyses were based on vitamin D supplementation, alcohol, smoking, BMI, diabetes, sleep, and outdoor exposure. Categorical variables were described by frequencies and compared with chi-squared tests.

**Results:**

During a median follow-up of 13.8 years, all cancer incidence was 5.1% (*n* = 2,614), with ovarian cancer at 0.3% (n = 176), breast cancer at 4.4% (*n* = 2,232), and uterine body cancer at 0.5% (*n* = 235). Low serum 25 (OH) D (50 nmol/L) was linked to increased risks of ovarian (HR: 1.457, 95% CI: 1.047–2.027) and uterine body cancer (HR: 1.372, 95% CI: 1.023–1.841). Each 10 nmol/L increase in 25 (OH) D reduced ovarian cancer risk (HR: 0.904, 95% CI: 0.835–0.979). Alcohol use and sleep <6 h were risk factors for ovarian and uterine cancer in those with low 25 (OH) D levels.

**Conclusion:**

Maintaining adequate serum 25 (OH) D levels is essential for overall health, reducing the risk of ovarian cancer, and potentially lowering susceptibility to uterine corpus cancer.

## Introduction

Vitamin D, a micronutrient, is primarily produced by the skin when exposed to sufficient ultraviolet B radiation from the sun, but can also be acquired from food or supplements (1, 2). The most common type of vitamin D found in the bloodstream is 25 (OH) D, with a half-life of around 2–3 weeks and maintaining stability. Therefore, assessing the concentration of circulating 25-hydroxyvitamin D is the most dependable way to determine a person’s vitamin D concentration and overall nutritional level of vitamin D (3). The active form of vitamin D 1,25 (OH)₂D₃ performs its physiological role when bound to the vitamin D receptor (VDR) (4). Vitamin D mainly functions to regulate calcium and phosphorus. In recent years, its potential functions in bone health, immune function, cardiovascular health, cancer prevention, etc. have attracted much attention (5–7). Preclinical research has found that vitamin D influences the proliferation, differentiation, and apoptosis of human cancer cells, potentially inhibiting carcinogenesis and slowing tumor progression by promoting cell differentiation and inhibiting metastasis (8–11).

Studies have shown that serum 25 (OH) D deficiency is very common in the population. The 2011–2014 US Health and Nutrition Survey found that around 70% of females lack vitamin D, with the likelihood of insufficient vitamin D concentration being highest in individuals between 20 and 39 years old and slightly lower in those 60 years old and older. Vitamin D deficiency is very common in premenopausal women (12). Numerous research has demonstrated a relationship between insufficient concentration of vitamin D and an elevated susceptibility to colorectal, prostate, and breast cancers in premenopausal women (13–16). Additional research conducted in Australia on patients with invasive ovarian cancer revealed that increased concentration of serum 25 (OH) D at the time of diagnosis were linked to improved survival rates (17). Prospective cohort studies have demonstrated that there is no significant correlation between serum 25 (OH) D levels and the incidence of endometrial cancer (18). Despite the progress made in researching the link between vitamin D and malignant tumors, studies on the effect of vitamin D deficiency on the risk of developing female-specific cancers in premenopausal women are still lacking. Further investigation is warranted to elucidate this potential association (19). Longitudinal monitoring of health outcomes in premenopausal women in prospective cohort studies examines the relationship between vitamin D levels and the development of female-specific cancers.

This study utilized data from the UK Biobank (UKB) to examine the association between serum 25 (OH) D levels and specific tumorigenesis in premenopausal women, exploring whether vitamin D may help prevent certain types of cancer in women before menopause and providing evidence to support public health guidelines.

## Methods

### Study design and participants

**Figure 1:**
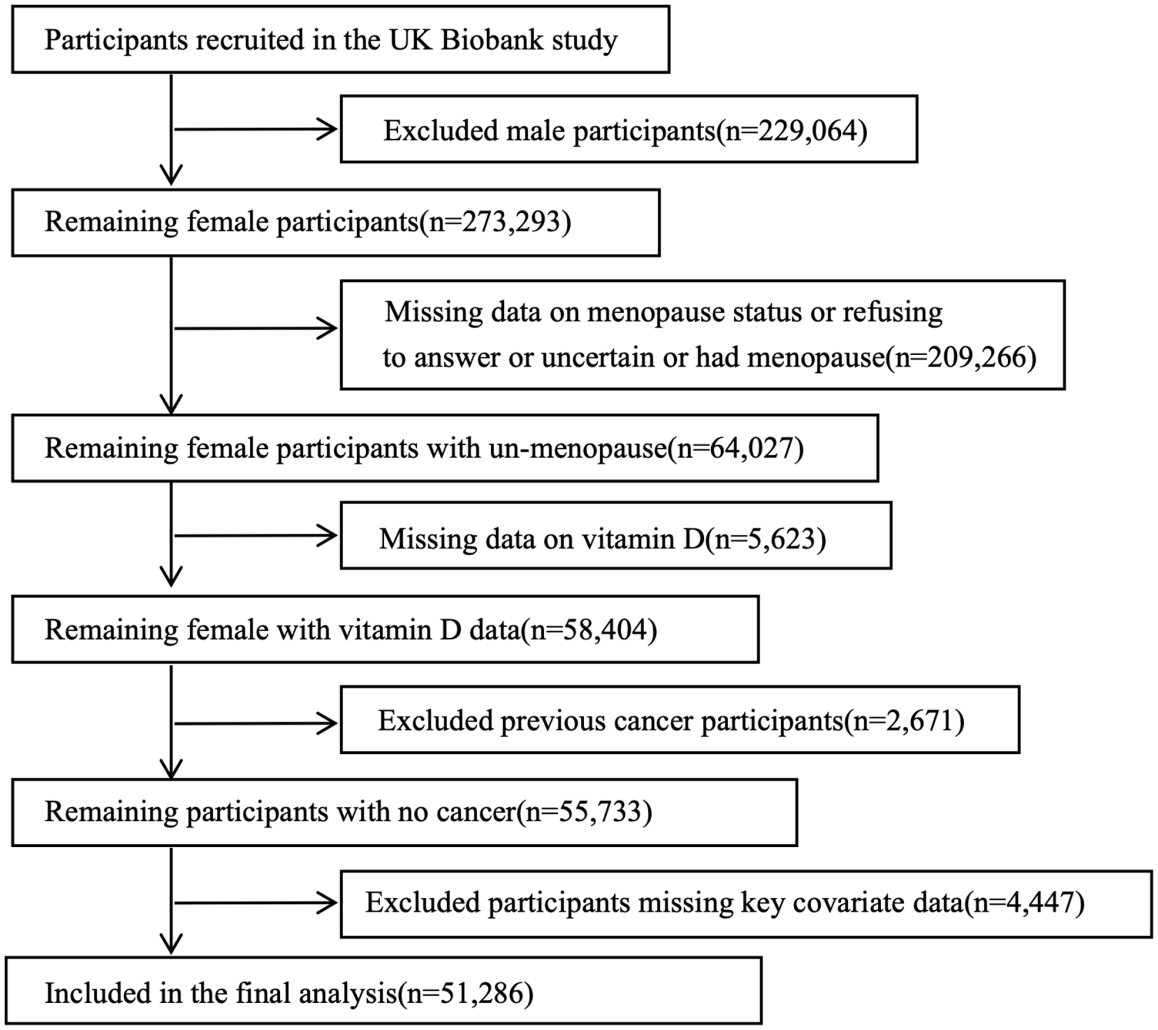
Flow chart showing the patient selection for the study.

The UKB is a prospective cohort study initiated by the UK government that collected more than 500,000 samples from within the Commonwealth between 2006 and 2010 from 22 assessment centers. Participants consisted of women between the ages of 37–73. Data collection included questionnaires, physical exams, and samples of blood, urine, and saliva. Health outcomes were abstracted using hospital records and cancer registries. The research was authorized by the Ethics Board, and all participants gave informed consent in an electronic questionnaire. The UK Biobank approved limited data access (application number 106397) for a research project centered on premenopausal females (aged 39–70 years) with initial serum 25 (OH) D levels recorded (n = 58,404). Exclusion criteria included a history of cancer (n = 2,671), missing key covariate data (n = 4,447), comprised age, TSO, TDI, VD_supplement, DM, drink, smoke, BMI, number of live births, OCP, HRT, sleep duration, milk type. Cancer status at baseline was determined through self-reported medical conditions and hospital inpatient data. Ultimately, the analytic cohort comprised 51,286 premenopausal women, including: (1) women with natural premenopausal status, and (2) HRT-treated women who maintained cyclic menstrual function prior to treatment initiation (Figure 1).

### Measurement of serum 25 (OH) D

Volunteer blood samples were obtained during the initial recruitment period from 2006 to 2010, subsequently processed, and preserved at −80 °C in liquid nitrogen. The density of serum 25 (OH) D was measured in nanomoles per liter (nmol/L) using a chemiluminescent immunoassay technique on the DiaSorin Liaison XL platform at the central laboratory facility of the UKB. This assay demonstrates a sensitivity range of 10–375 nmol/L for detecting vitamin D, enabling precise assessment of vitamin D concentrations across a broad range. For in-depth information on how the assay performs, visit the UK Biobank website (20). The overall variation coefficient for serum 25 (OH) D ranged from 5.04 to 6.14%, with external quality control at 100% (21). Previous research has confirmed that normal, deficient, and low vitamin D concentrations are defined as ≥50 nmol/L, 30–50 nmol/L, and <30 nmol/L (22), respectively.

### Determination of gynecologic malignant tumor cases

Hospital records from inpatient databases in England, Scotland, and Wales dating back to 1998 were accessed for this study. Data on female-specific malignancies were collected by the National Cancer Registry from regional cancer centers across the UK. Malignancy status for ovarian cancer (C56), breast cancer (C50, D05), and uterine body cancer (C54, D07.0) was classified using ICD-10 codes. Cervical malignancies were excluded from the analysis due to the small number of cervical cancer cases. If a participant had multiple gynecologic malignancies, they were counted as a single case in all cancer cases. The date of disease onset was defined as the first recorded date. Follow-up began from the baseline recruitment date until the onset of disease or the end of follow-up.

### Covariate assessment

Covariate data were obtained from touchscreen questionnaires completed at recruitment, covering sociodemographic factors (age, Townsend Deprivation Index [TDI]), lifestyle factors (history of alcohol use, history of smoking, sleep duration, time spent outdoors [TSO], milk type used), female-specific factors (number of live births, oral contraceptive [OCP], hormone replacement therapy [HRT]), health-related outcomes (Diabetes Mellitus [DM]) and health history (vitamin D supplement). The trained nurses meticulously collected data on individuals’ weight and height measurements at recruitment, utilizing these figures to compute their Body Mass Index (BMI, kg/m^2^). TDI is an indicator for measuring the socio-economic environment (23). TSO was characterized by the average amount of time spent outdoors each day during both the summer and winter seasons. The utilization of Vitamin D supplementation encompasses the consumption of both vitamin D and multivitamins.

### Statistical analysis

The study calculated medians (interquartile spacing) for continuous variables and employed the Wilcoxon rank sum test to assess differences in baseline characteristics between cohorts with serum 25 (OH) D concentration ≥50 nmol/L and those with concentration <50 nmol/L. Categorical variables were described and compared using frequencies (percentages) and the chi-squared test.

We analyzed the connection between serum 25 (OH) D density and the incidence of female-specific malignancies using a multivariable Cox regression model, adjusting for age, TSO, TDI, VD_supplement, DM, drink, smoke, BMI, number of live births, OCP, HRT, sleep duration, milk type. The serum 25 (OH) D was included in the Cox regression model in three forms respectively: continuous variables (per 10 nmol/L increment), binary classification (50 nmol/L vs. ≥ 50 nmol/L), and trinary classification (30–50 nmol/L or <30 nmol/L vs. ≥ 50 nmol/L). To evaluate potential nonlinear associations between gynecologic malignancy incidence and serum 25 (OH) D concentration, we utilized restricted cubic splines (RCS) with three knots.

Sensitivity analyses were performed using propensity score matching (PSM) and overlap weight (OW) propensity score weighting models to compare the incidence of female-specific malignancies between groups with serum 25 (OH) D concentration <50 nmol/L and ≥50 nmol/L (24). The propensity score (PS) was estimated by using logistic regression including all baseline variables. PSM was performed using the nearest-neighbor method, with a caliper set at 0.01 standard deviations of the logit-transformed estimated propensity score value, along with the matching ratio. The PSM was conducted by using the “MatchIt” R package. The OW was calculated by using the “PSW” R package. ASMDs were computed to assess the effectiveness of PS matching and weighting in minimizing disparities between the two groups. The covariate was viewed as being balanced, given that its ASMD was less than 0.1 (25). Subgroup analyses were performed based on vitamin D supplement, DM, drinking status, smoking status, BMI, sleep duration, and time spent outdoors.

All statistical analyses were performed utilizing the R software version 4.0.3. All *p* values reported were considered statistically significant if they were less than 0.05, with all tests being two-sided.

## Results

### Baseline characteristics of subjects

Table 1 displays the baseline demographics of the 51,286 individuals involved in the study. The median age was 46 years (IQR 43–49), the median TSO was 2 h, and the median TDI was −1.98. Approximately 26.5% of women took vitamin D supplements. Only a small minority of women (3.3%) are diagnosed with diabetes. Most women consumed alcohol currently (93%), few smoked, and the median BMI was 25.18 kg/m^2^, the median number of live births was 2, with most women having used OCPs. Only 3.5% had used HRT, and the median sleep duration was 7 h. Low or deficient serum 25 (OH) D density were identified in 30,698 volunteers, representing 59.8% of the total sample. Females with elevated levels were typically thinner, non-smokers, less likely to have diabetes, had lower TDI scores, higher alcohol intake, used OCPs and HRT, and regularly consumed vitamin D supplements (all *p* < 0.001, see Table 1 and Supplementary Table S1).

**Table 1:** Baseline characteristics of study participants by vitamin D levels.

	Overall *N* = 51,286	Original cohort				PSM cohort			
		Normal (VD ≥ 50 nmol/L) *N* = 20,588	Deficiency or Low (VD < 50 nmol/L) *N* = 30,698	*p*	ASMD	Normal (VD ≥ 50 nmol/L) *N* = 19,153	Deficiency or Low (VD < 50 nmol/L) *N* = 19,153	*p*	ASMD
Age, median [IQR]	46.00 [43.00, 49.00]	46.00 [43.00, 49.00]	46.00 [43.00, 49.00]	0.026	0.009	46.00 [43.00, 49.00]	46.00 [43.00, 49.00]	0.032	0.007
TSO, median [IQR]	2.00 [1.25, 3.00]	2.00 [1.50, 3.00]	2.00 [1.25, 3.00]	<0.001	0.151	2.00 [1.50, 3.00]	2.00 [1.25, 3.00]	<0.001	0.005
TDI, median [IQR]	−1.98 [−3.57, 0.71]	−2.32 [−3.73, 0.02]	−1.69 [−3.43, 1.13]	<0.001	0.206	−2.23 [−3.68, 0.18]	−2.23 [−3.70, 0.25]	0.686	0.003
VD_supplement, *n* (%)	13,580 (26.5)	6,797 (33.0)	6,783 (22.1)	<0.001	0.246	5,579 (29.1)	5,533 (28.9)	0.612	0.005
DM, *n* (%)	1703 (3.3)	413 (2.0)	1,290 (4.2)	<0.001	0.127	408 (2.1)	425 (2.2)	0.575	0.006
Drinking, *n* (%)				<0.001	0.165			0.877	0.005
Never	2,210 (4.3)	509 (2.5)	1701 (5.5)			509 (2.7)	495 (2.6)		
Previous	1,371 (2.7)	463 (2.2)	908 (3.0)			450 (2.3)	443 (2.3)		
Current	47,705 (93.0)	19,616 (95.3)	28,089 (91.5)			18,194 (95.0)	18,215 (95.1)		
Smoke, *n* (%)				<0.001	0.052			0.779	0.007
Never	33,171 (64.7)	13,136 (63.8)	20,035 (65.3)			12,267 (64.0)	12,201 (63.7)		
Previous	13,130 (25.6)	5,538 (26.9)	7,592 (24.7)			5,065 (26.4)	5,118 (26.7)		
Current	4,985 (9.7)	1914 (9.3)	3,071 (10.0)			1821 (9.5)	1834 (9.6)		
BMI, median [IQR]	25.18 [22.68, 28.75]	24.49 [22.32, 27.39]	25.73 [22.99, 29.76]	<0.001	0.322	24.68 [22.45, 27.65]	24.68 [22.39, 27.83]	0.980	0.008
Number of live births, median [IQR]	2.00 [0.00, 2.00]	2.00 [1.00, 2.00]	2.00 [0.00, 2.00]	<0.001	0.071	2.00 [1.00, 2.00]	2.00 [1.00, 2.00]	0.475	0.003
OCP, *n* (%)	45,762 (89.2)	18,968 (92.1)	26,794 (87.3)	<0.001	0.160	17,560 (91.7)	17,588 (91.8)	0.616	0.005
HRT, *n* (%)	1776 (3.5)	804 (3.9)	972 (3.2)	<0.001	0.040	691 (3.6)	700 (3.7)	0.827	0.003
Sleep duration, median [IQR]	7.00 [7.00, 8.00]	7.00 [7.00, 8.00]	7.00 [7.00, 8.00]	<0.001	0.078	7.00 [7.00, 8.00]	7.00 [7.00, 8.00]	0.488	0.003
Milk type used, *n* (%).	5,068 (9.9)	1949 (9.5)	3,119 (10.2)	0.010	0.023	1815 (9.5)	1841 (9.6)	0.664	0.005
All Cancer, *n* (%)	2,614 (5.1)	1,005 (4.9)	1,609 (5.2)	0.072	0.016	932 (4.9)	993 (5.2)	0.161	0.015
Ovary cancer, *n* (%)	176 (0.3)	54 (0.3)	122 (0.4)	0.013	0.024	48 (0.3)	73 (0.4)	0.029	0.023
Breast cancer, *n* (%)	2,232 (4.4)	897 (4.4)	1,335 (4.3)	0.982	<0.001	831 (4.3)	844 (4.4)	0.764	0.003
Uterine body cancer, *n* (%)	235 (0.5)	67 (0.3)	168 (0.5)	<0.001	0.034	65 (0.3)	87 (0.5)	0.088	0.018

TSO, Time spent outdoors (hours); TDI, Townsend deprivation index; DM, Diabetes Mellitus; OCP, Oral contraceptive; HRT, hormone replacement therapy; PSM, Propensity score matching; ASMD, Absolute standardized mean differences; VD, Serum 25 (OH) D; BMI, body mass index.

Before conducting PSM, Table 1 and Supplementary Figure S1A illustrate significant disparities in the distribution of propensity scores between the low and deficient serum 25 (OH) D density groups, compared to the reference group (sufficient 25 (OH) D ≥ 50 nmol/L). Participants showed notable variations in their TSO, TDI, BMI, alcohol consumption, vitamin D intake, DM and use of oral contraceptive pills (ASMD>0.1). Following PSM, 19,153 pairs of cases were matched based on low and deficient serum 25 (OH) D density compared with normal levels, with no significant variations in the mentioned factors observed between the two matched groups (ASMD<0.1).

### Serum 25 (OH) D levels and the risk of female-specific malignancies

Over a 13.8-year following period, the incidence ratio of all cancers was 5.1% (*n* = 2,614), with ovarian cancer at 0.3% (*n* = 176), breast cancer at 4.4% (*n* = 2,232), and uterine body cancer at 0.5% (*n* = 235) (Table 1). Participants who had serum 25 (OH) D density lower than 50 nmol/L exhibited an increased risk of developing ovarian cancer (adjusted hazard ratio [HR] 1.457, 95% confidence interval [CI] 1.047–2.027) and uterine body cancer (adjusted HR 1.372, 95% CI 1.023–1.841) when compared to individuals with serum 25 (OH) D density ≥ 50 nmol/L. However, there was no statistically significant difference in the risk of all cancers and breast cancer (Table 2).

**Table 2:** Primary endpoint analysis of vitamin D levels and female-specific cancers.

	All Cancer		Ovary cancer^		Breast cancer		Uterine body cancer^	
	HR (95% CI)	P	HR (95% CI)	P	HR (95% CI)	P	HR (95% CI)	P
Vitamin D, per 10 nmol/L increase ^*^	0.979 (0.96–0.998)	0.0279	0.904 (0.835–0.979)	0.0129	0.989 (0.969–1.009)	0.2845	0.945 (0.882–1.013)	0.1095
Low or Deficiency (50 nmol/L) vs. Normal (≥50 nmol/L)								
Multivariable Cox regression ^*^	1.066 (0.983–1.157)	0.1219	1.457 (1.047–2.027)	0.0257	1.014 (0.929–1.107)	0.7532	1.372 (1.023–1.841)	0.0347
Propensity score matching ^#^	1.069 (0.977–1.169)	0.1454	1.521 (1.056–2.189)	0.0241	1.018 (0.925–1.120)	0.7213	1.337 (0.969–1.844)	0.0767
OW weighting ^$^	1.065 (0.982–1.156)	0.1279	1.435 (1.031–1.998)	0.0324	1.012 (0.928–1.105)	0.7834	1.36 (1.013–1.826)	0.0408
Three group compare ^*^								
Deficiency (<30 nmol/L) vs. Normal (≥50 nmol/L)	1.083 (0.978–1.199)	0.1237	1.668 (1.131–2.461)	0.0099	1.023 (0.916–1.143)	0.6859	1.35 (0.952–1.914)	0.0926
Low (30–50 nmol/L) vs. Normal (≥50 nmol/L)	1.056 (0.965–1.156)	0.2389	1.326 (0.919–1.913)	0.1315	1.009 (0.915–1.111)	0.8634	1.387 (1.009–1.906)	0.0437

*The hazard ratios and *p-*values were obtained by using multivariable Cox regression with adjusting for age, TSO, TDI, VD_supplement, DM, drink, smoke, BMI, number of live births, OCP, HRT, sleep duration, milk type used. # The hazard ratios and p values were obtained by using Cox regression based on propensity score matched cohort. $ The hazard ratios and *p-*values were obtained by using Cox regression with overlap weights weighting. ^ For the analysis of ovarian cancer, 63 patients who had undergone bilateral oophorectomy at enrollment were excluded. Similarly, for the analysis of uterine body cancer, 218 patients who had undergone hysterectomy at enrollment were excluded. TSO, Time spent outdoors (hours); TDI, Townsend deprivation index; DM, Diabetes Mellitus; OCP, Oral contraceptive; HRT, hormone replacement therapy; BMI, body mass index.

**Figure 2:**
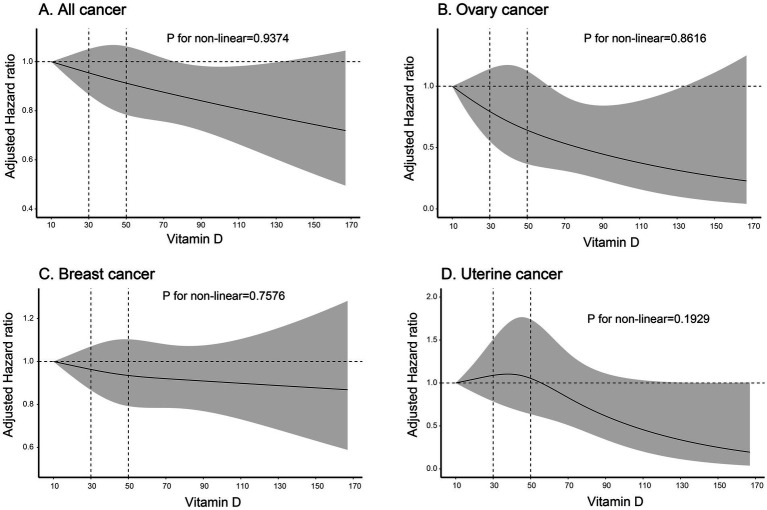
Risk of cancer incidence according to Vitamin D. The solid line indicates AHRs for any vitamin D, with corresponding 95% CIs indicated by the shaded area. Vitamin D, Serum 25 (OH) D; AHRs, adjusted hazard ratios. **(A)** Association between vitamin D levels and all cancer risk; **(B)** Association between vitamin D levels and ovary cancer risk; **(C)** Association between vitamin D levels and breast cancer risk; **(D)** Association between vitamin D levels and uterine cancer risk.

In sensitivity analyses, baseline characteristics of covariates after PSM and OW weighting are shown in Supplementary Figure S1B. In both the PSM (*n* = 38,306) and OW weighted cohorts, serum 25 (OH) D concentrations <50 nmol/L were associated with an increased risk of ovarian cancer, with HR of 1.521 (95% CI: 1.056–2.189) and 1.435 (95% CI: 1.031–1.998), respectively, when compared to concentrations ≥50 nmol/L. A similar association was observed for uterine body cancer in the OW cohort, whereas no such association was identified in the PSM cohort. Furthermore, no significant associations were observed between serum 25 (OH) D concentrations and the risks of breast cancer or all cancer in either cohort (Table 2). When serum 25 (OH) D concentrations were categorized into three groups (30, 30–50, and ≥50 nmol/L), individuals with serum 25 (OH) D concentrations <30 nmol/L had an increased risk of ovarian cancer (adjusted HR 1.668, 95% CI 1.131–2.461), while those with concentrations between 30 and 50 nmol/L had an increased risk of uterine body cancer (adjusted HR 1.387, 95% CI 1.009–1.906), compared to those with normal serum 25 (OH) D levels (≥50 nmol/L) (Table 2). Table 2 demonstrates that a rise of 10 nmol/L in serum 25 (OH)D levels was linked to a decreased risk of ovarian malignant tumors (adjusted HR 0.904, 95% CI 0.835–0.979) and all malignant tumors (adjusted HR 0.979, 95% CI 0.96–0.998). The dose–response relationships showed no evidence of non-linearity (all P-nonlinear>0.05) (Figure 2), supporting linear associations as confirmed by significant risk reductions per 10 nmol/L 25 (OH) D increase for all and breast cancer (both *p* < 0.05).

### Accumulated cancer occurrence based on serum 25 (OH) D levels

**Figure 3:**
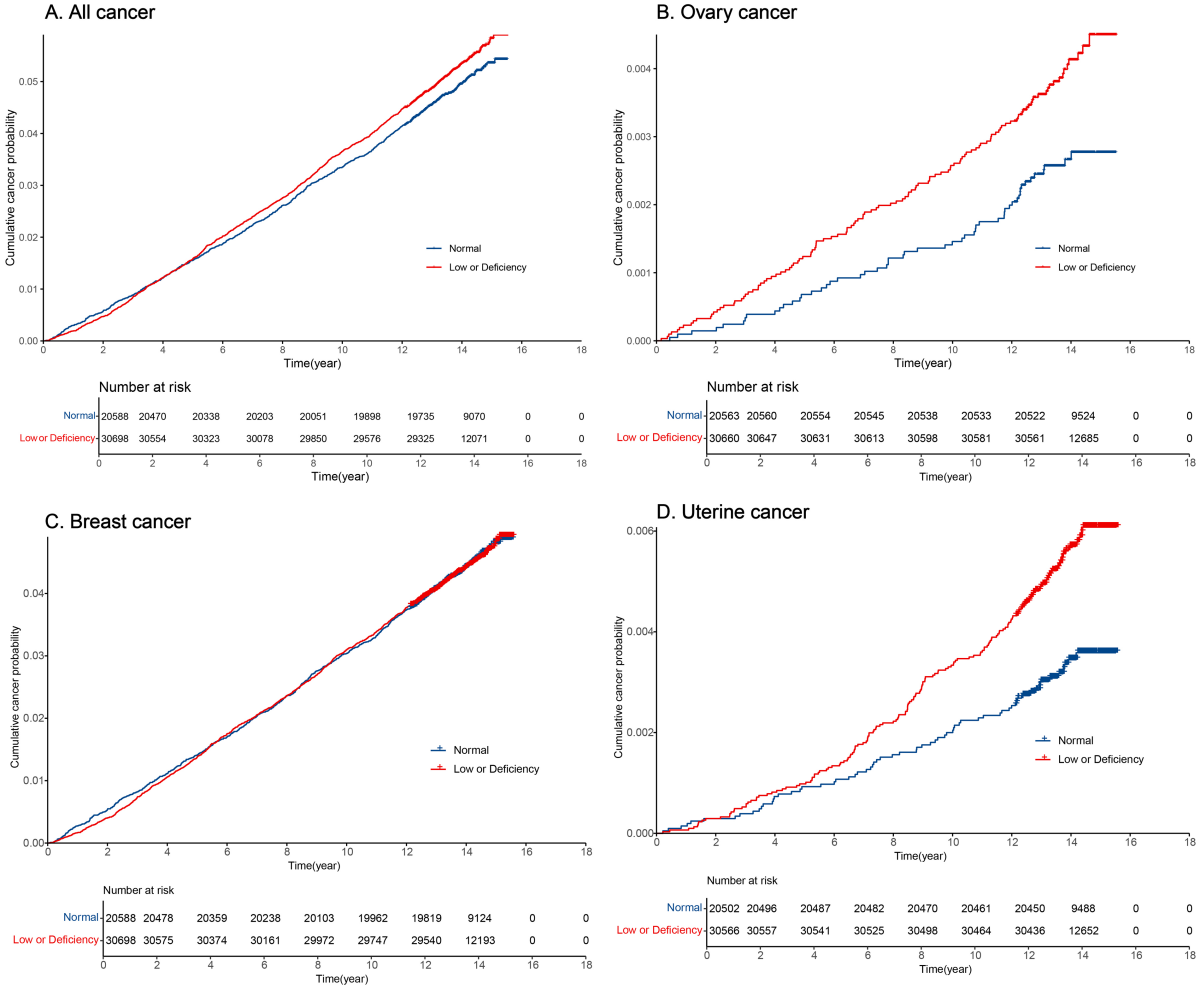
Cumulative incidence of cancer over time in normal and low or deficiency groups. **(A)** Cumulative incidence of all cancer over time; **(B)** Cumulative incidence of ovary cancer over time; **(C)** Cumulative incidence of breast cancer over time; **(D)** Cumulative incidence of uterine cancer over time.

Supplementary Table S2 displays the cumulative incidence rates for all cancer, ovarian cancer, breast cancer, and uterine body cancer over 5, 10, and 15 years. Figure 3 illustrates the cumulative incidence over time using a cumulative incidence function. Overall, the cumulative incidence of malignancies increased steadily over time. Individuals with serum 25 (OH) D concentrations <50 nmol/L had a notably higher cumulative incidence of ovarian cancer (Figure 3B) and uterine body cancer (Figure 3D) than those with levels ≥50 nmol/L, showing statistical significance (*p* < 0.05). Ovarian cancer showed different cumulative incidence rates over 5, 10, and 15 years based on serum 25 (OH) D density, for levels ≥50 nmol/L with ratios of 0.7‰, 1.5‰, 2.8‰, and <50 nmol/L with the ratio of 1.2‰, 2.6‰, and 4.5‰, respectively. In cases of uterine body cancer, the 5-year cumulative incidence rates for serum 25 (OH) D levels ≥50 nmol/L and <50 nmol/L were 0.9‰ and 1‰. The 10-year cumulative incidence rates were 2‰ and 3.3‰, respectively, while the 15-year cumulative incidence rates were 3.6‰ and 6.1‰, respectively (Supplementary Table S2). For both all cancers (Figure 3A) and breast cancer (Figure 3C), there were no notable variances in cumulative incidence rates when comparing serum 25 (OH) D density <50 nmol/L and ≥50 nmol/L (*p* > 0.05).

### Subgroup analysis

The following were revealed through subgroup analyses that considered vitamin D supplementation, alcohol consumption, smoking status, BMI, DM, sleep duration, and TSO.

**Figure 4:**
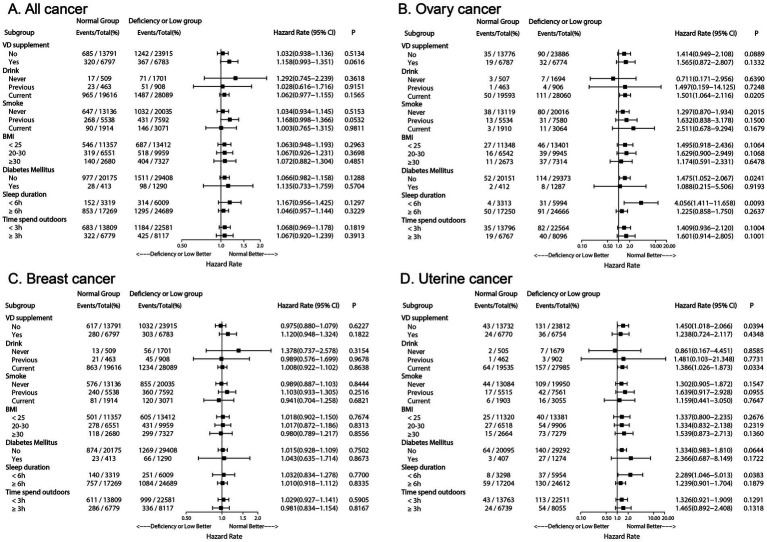
Forest plot depicting adjusted hazard ratios of Low or Deficiency (50) group versus Normal (≥50) group for all cancer and each cancer incidence risk in subgroup population. **(A)** Forest plot of all cancer risk in subgroup population; **(B)** Forest plot of ovary cancer risk in subgroup population; **(C)** Forest plot of breast cancer risk in subgroup population; **(D)** Forest plot of uterine cancer risk in subgroup population. VD, Vitamin D; BMI, body mass index.

In the subgroup analysis of ovarian cancer (Figure 4B), serum density of 25 (OH) D was found to be statistically significant in current drinkers, but not in previous or non-drinkers. Current drinkers with serum 25 (OH) D concentration below 50 nmol/L had a 50.1% higher risk of ovarian cancer compared to those in the standard serum 25 (OH) D category (HR 1.501; 95% CI 1.064–2.116; *p* = 0.0205). Among individuals with sleep durations less than 6 h, having serum 25 (OH) D levels below 50 nmol/L was identified as a risk factor for ovarian cancer in the subgroup analysis on sleep duration (HR 4.056; 95% CI 1.411–11.658; *p* = 0.0093), while this association was not observed in individuals with sleep durations of 6 h or more. The subgroup analysis for DM showed that, compared to individuals with normal serum 25 (OH) D levels, non-diabetic individuals with serum 25 (OH) D concentrations below the normal range had a 47.5% increased risk of ovarian cancer (HR 1.475; 95% CI: 1.052–2.067; *p* = 0.0241).

A similar trend was observed for uterine body cancer (Figure 4D). Compared to women with adequate serum 25 (OH) D concentrations, women who did not supplement with vitamin D, currently drank alcohol, and slept less than 6 h had an increased likelihood of developing uterine body cancer if their serum 25 (OH) D concentrations were below 50 nmol/L (*p* < 0.05).

In the analyses for all cancers (Figure 4A) and breast cancer (Figure 4C), no significant differences were observed across subgroups (*p* > 0.05).

## Discussion

### Principal findings

Analysis of data from the UK Biobank over a 13.8-year follow-up period indicated that higher serum 25 (OH) D concentrations in premenopausal women were associated with a reduced incidence of ovarian cancer and potentially linked to a lower incidence of uterine body cancer. In individuals with a sleep duration of less than 6 h and those who currently consume alcohol, serum 25 (OH) D concentrations below 50 nmol/L were associated with a significantly increased risk of ovarian and uterine body cancer compared to those with sufficient serum 25 (OH) D levels.

### Results in the context of what is known

Circulating 25 (OH) D serves as the primary biomarker of vitamin D status, reflecting both endogenous synthesis and dietary intake. Limited research has been conducted on the correlation between levels of circulating 25 (OH) D and the likelihood of developing tumors specific to females in women before menopause (26). Prospective research has found an inverse relationship between circulating vitamin D concentrations and the risk of postmenopausal breast cancer, but this dose–response interaction was not observed in premenopausal women (27). A prospective study found that the predicted serum 25 (OH) D concentration was not related to the incidence rate of endometrial cancer (18). Our ovarian cancer findings align with a Canadian case–control study (OR = 0.72 per 20 nmol/L increase) (28).

A Mendelian randomization (MR) study in European populations reported an inverse association between genetically predicted lower serum 25 (OH) D levels and ovarian cancer risk (29). However, other MR studies found no significant associations between vitamin D concentrations and breast, ovarian, endometrial, or overall cancer risks (30–32). These null findings may reflect limited statistical power to detect subtype-specific effects (e.g., in high-grade serous ovarian cancer).

### Potential biological plausibility

1α,25 (OH) 2D3 can promote the production of antimicrobial peptides, suppress excessive inflammation to prevent tissue damage, bind to VDR in monocytes, induce the expression of the cathelicidin antimicrobial peptide (hCAP), and enhance bactericidal activity (33). Analyses of online databases of 33 human cancers have found increased expression of VDR in invasive breast cancer, serous ovarian adenocarcinoma, and endometrial adenocarcinoma. 1α,25 (OH) 2D3 influences gene transcription after binding to the VDR (4). In breast cancer cells, 1α,25 (OH) 2D3 has been found to inhibit tumor proliferation by binding to VDR, In ovarian cancer cells, 1,25 (OH) 2D3 upregulates p27 protein levels, inhibits cyclin E/CDK2 activity, leading to cell cycle arrest at G1 phase and suppression of proliferation (34). Vitamin D counteracts the WNT signaling pathway to suppress genes involved in tumor growth, metastasis and angiogenesis (35). Serum 25 (OH) D levels >50 nmol/L may reduce cancer risk in high-risk populations through the aforementioned mechanisms. VDR gene polymorphisms may influence the anticancer efficacy of vitamin D.

### Clinical implications

We did not observe a significant association between serum 25 (OH) D levels and breast cancer risk across various subgroups, a result that differs from the significant associations found for ovarian and uterine body cancers. In a 5-year follow-up study of postmenopausal women, Marie K. Dam reported that alcohol intake was associated with an increased risk of breast cancer (36). However, in the current study, no differences in breast cancer incidence were observed in the subgroup of current alcohol consumers based on serum 25 (OH) D levels. This may be because breast cancer is influenced by a variety of genetic, hormonal, and environmental factors. The impact of alcohol on breast cancer risk is thought to be mediated through pathways such as estrogen metabolism or oxidative stress (37), which may not directly relate to serum vitamin D levels. Additionally, Furthermore, breast cancer is a heterogeneous disease, with different subtypes potentially responding differently to vitamin D, making it more challenging to identify a clear association (38).

Modern medicine identifies diabetes and obesity as high-risk factors for endometrial cancer. In this study, subgroup analyses based on diabetes status and BMI revealed no significant association between serum 25 (OH) D levels and the risk of uterine body cancer, consistent with the findings of a case–control study conducted in 2010 (39). Circulating serum 25 (OH) D density is connected to various important factors. TSO has a strong positive correlation with levels of circulating serum 25 (OH) D (40). Sunlight exposure induces the production of previtamin D3, which is subsequently converted to vitamin D3 through isomerization (41). The longer the outdoor time, the higher the bioavailability of vitamin D. In this study, the majority of individuals with outdoor exposure time exceeding 3 h had insufficient serum 25 (OH) D levels, which may be related to lower vitamin D bioavailability in this population or insufficient ultraviolet exposure during the winter months.

### Research implications

The findings of this research have important implications for public health and clinical practice, particularly highlighting the high prevalence of vitamin D deficiency in premenopausal women. This highlights the importance of implementing specific measures to enhance serum 25 (OH) D density in this demographic (42). Public health initiatives may be necessary to educate the public on the significance of vitamin D, as well as provide recommendations for vitamin supplements and safe exposure to sunlight (19, 43). Moreover, the connection between serum 25 (OH) D density and the occurrence of ovarian and uterine corpus cancer underscores the possible importance of vitamin D in preventing cancer. While the exact processes are still not completely clear, the current data suggests that ensuring adequate vitamin D levels could be a simple and effective way to reduce the likelihood of developing these specific cancers in women before menopause.

### Strengths and limitations

This study has several strengths, including a large sample size, robust statistical analysis, and comprehensive adjustment for potential confounders, but it also has its limitations. Specifically, this is an observational study, so causal relationships cannot be inferred. The association between serum 25 (OH) D concentrations and cancer risk may be influenced by unmeasured confounding factors, including participants’ race and the season of blood sample collection. Additionally, this study relied on a single measurement of serum 25 (OH) D, which may not fully reflect long-term vitamin D levels or changes in individual lifestyle factors that could impact vitamin D metabolism. Further longitudinal studies are needed to validate these findings and explore the causal mechanisms between vitamin D and cancer risk. Recognizing the complexity of the relationship between cancer and vitamin D is essential, as it is affected by various factors (44, 45). Our study offers valuable insights; However, more studies are needed to validate these discoveries and illustrate the biological mechanisms behind them.

## Conclusion

This study demonstrates that insufficient serum 25 (OH) D levels are associated with an increased risk of ovarian cancer and potentially endometrial cancer, particularly among individuals with alcohol consumption and sleep duration less than 6 h. Our findings further substantiate the importance of maintaining adequate vitamin D levels for overall health and reducing potential risks of specific cancers. Public health policies should prioritize optimizing vitamin D status, while further research is needed to elucidate the complex relationship between vitamin D and cancer risk, which may lead to improved prevention and treatment strategies for premenopausal women’s malignancies. Although our study supports maintaining sufficient vitamin D status for cancer prevention, individualized risk–benefit assessments remain crucial, especially for patients with a history of nephrolithiasis or granulomatous disorders.

## Data Availability

The raw data supporting the conclusions of this article will be made available by the authors, without undue reservation.
