# Supplementary material for: Serum 25 (OH) D levels and risk of female-specific cancer in premenopausal women: a prospective study
Source: Front Nutr. 2025 Sep 15;12:1617565. doi: 10.3389/fnut.2025.1617565 (PMC12477915; doi:10.3389/fnut.2025.1617565)
Supplement: Supplementary file 1 [file Image_1.pdf]

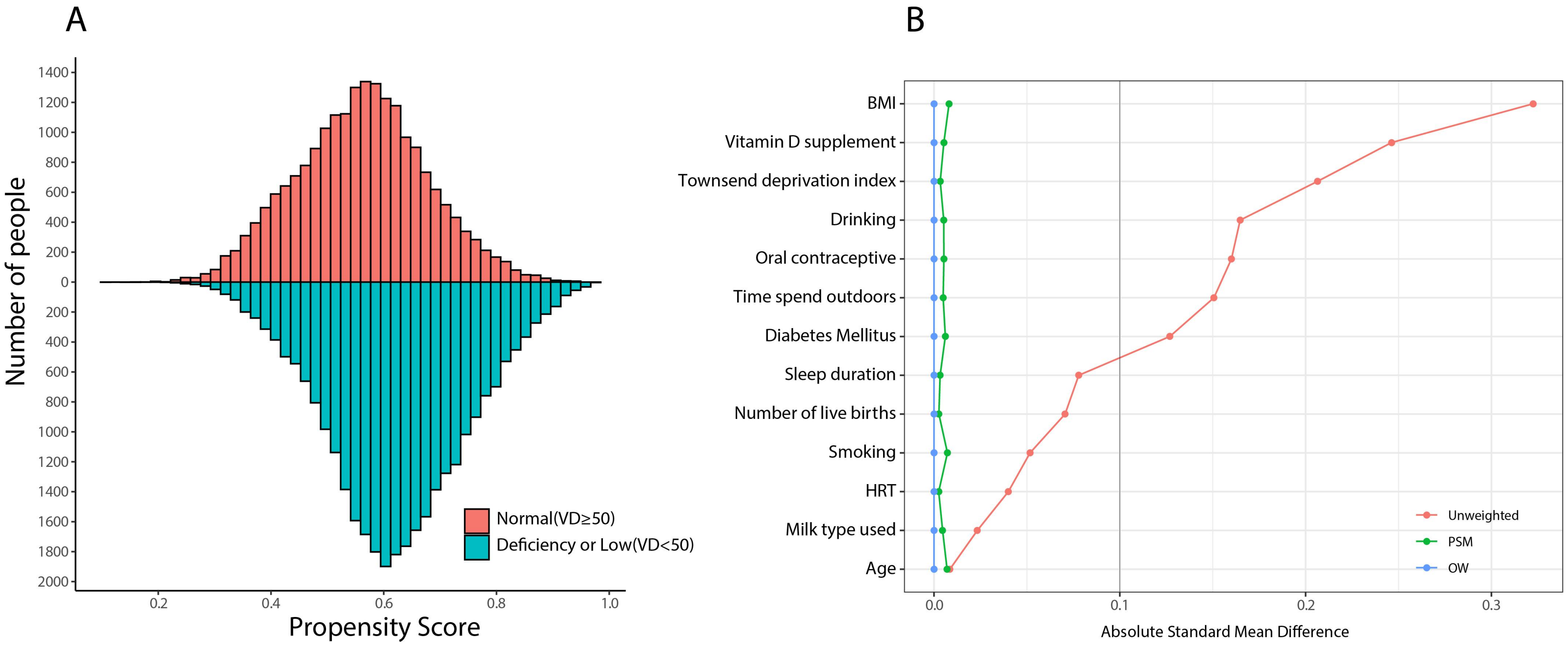

Figure s1 The distribution of propensity score and balance of covariates between two groups.

A. the distribution of propensity score among two groups. B. the absolute standard mean difference of each covariate between two group in three target population (unweighted/original cohort, propensity score matched cohort, overlap weights weighted cohort). The ASMD criterion for covariate unbalance set to 0.1.

HRT, hormone replacement therapy; BMI, body mass index; PSM, Propensity score matching; OW, overlap weights.
