# Supplementary material for: Serum 25 (OH) D levels and risk of female-specific cancer in premenopausal women: a prospective study
Source: Front Nutr. 2025 Sep 15;12:1617565. doi: 10.3389/fnut.2025.1617565 (PMC12477915; doi:10.3389/fnut.2025.1617565)
Supplement: Supplementary file 2 [file Table_1.pdf]

Supplemental:

Table S1 Baseline Charactristic

	Overall N=51539	Vitamin D groups			p
		Normal(VD⩾50 nmol/L) N=20588	Deficiency(VD<30 nmol/L) N=12749	Low(VD 30-50 nmol/L) N=17949	
Age, median[IQR]	46.00 [43.00, 49.00]	46.00 [43.00, 49.00]	46.00 [43.00, 49.00]	46.00 [43.00, 49.00]	0.022
TSO, median[IQR]	2.00 [1.25, 3.00]	2.00 [1.50, 3.00]	1.75 [1.25, 2.75]	2.00 [1.25, 3.00]	<0.001
TDI, median[IQR]	-1.98 [-3.57, 0.71]	-2.32 [-3.73, 0.02]	-1.24 [-3.23, 1.70]	-1.96 [-3.55, 0.68]	<0.001
VD_supplement, n(%)	13580 (26.5)	6797 (33.0)	2184 (17.1)	4599 (25.6)	<0.001
DM, n(%)	1703 (3.3)	413 (2.0)	710 (5.6)	580 (3.2)	<0.001
Drinking, n(%)					<0.001
Never	2210 (4.3)	509 (2.5)	991 (7.8)	710 (4.0)	
Previous	1371 (2.7)	463 (2.2)	446 (3.5)	462 (2.6)	
Current	47705 (93.0)	19616 (95.3)	11312 (88.7)	16777 (93.5)	
Smoke, n(%)					<0.001
Never	33171 (64.7)	13136 (63.8)	8401 (65.9)	11634 (64.8)	
Previous	13130 (25.6)	5538 (26.9)	2908 (22.8)	4684 (26.1)	
Current	4985 (9.7)	1914 (9.3)	1440 (11.3)	1631 (9.1)	
BMI, median[IQR]	25.18 [22.68, 28.75]	24.49 [22.32, 27.39]	26.06 [23.12, 30.50]	25.52 [22.92, 29.26]	<0.001
Number of live births, median[IQR]	2.00 [0.00, 2.00]	2.00 [1.00, 2.00]	2.00 [0.00, 2.00]	2.00 [0.00, 2.00]	<0.001
OCP, n(%)	45762 (89.2)	18968 (92.1)	10775 (84.5)	16019 (89.2)	<0.001
HRT, n(%)	1776 (3.5)	804 (3.9)	388 (3.0)	584 (3.3)	<0.001
Sleep duration, median[IQR]	7.00 [7.00, 8.00]	7.00 [7.00, 8.00]	7.00 [7.00, 8.00]	7.00 [7.00, 8.00]	<0.001
Milk type used, n(%).	5068 (9.9)	1949 (9.5)	1327 (10.4)	1792 (10.0)	0.017
All Cancer, n(%)	2614 (5.1)	1005 (4.9)	677 (5.3)	932 (5.2)	0.173

Ovary cancer, n(%)	176 (0.3)	54 (0.3)	58 (0.5)	64 (0.4)	0.013
Breast cancer, n(%)	2232 (4.4)	897 (4.4)	553 (4.3)	782 (4.4)	0.996
Uterine cancer, n(%)	235 (0.5)	67 (0.3)	74 (0.6)	94 (0.5)	0.001

TSO, Time spent outdoors (hours); TDI, Townsend deprivation index; DM, Diabetes Mellitus; OCP, Oral contraceptive; HRT, hormone replacement therapy;VD, Serum 25(OH)D; BMI, body mass index.
