# Supplementary material for: Serum 25 (OH) D levels and risk of female-specific cancer in premenopausal women: a prospective study
Source: Front Nutr. 2025 Sep 15;12:1617565. doi: 10.3389/fnut.2025.1617565 (PMC12477915; doi:10.3389/fnut.2025.1617565)
Supplement: Supplementary file 3 [file Table_2.pdf]

Table S2 The cumulative incidence rates of malignancies

| | Normal | Deficiency or Low |
|---|---|---|
| gynecologic malignancies | | |
| All cancer | | |
| 5 years | 0.0156(0.0139-0.0173) | 0.0158(0.0144-0.0172) |
| 10 years | 0.0335(0.0311-0.036) | 0.0365(0.0344-0.0386) |
| 15 years | 0.0536(0.05-0.0572) | 0.0584(0.0551-0.0616) |
| ovarian cancer | | |
| 5 years | 0.0007(0.0003-0.001) | 0.0012(0.0008-0.0016) |
| 10 years | 0.0015(0.0009-0.002) | 0.0026(0.002-0.0031) |
| 15 years | 0.0028(0.002-0.0035) | 0.0045(0.0036-0.0054) |
| breast cancer | | |
| 5 years | 0.0141(0.0125-0.0157) | 0.0137(0.0124-0.015) |
| 10 years | 0.0304(0.0281-0.0327) | 0.031(0.029-0.0329) |
| 15 years | 0.048(0.0445-0.0514) | 0.0484(0.0454-0.0514) |
| uterine body cancer | | |
| 5 years | 0.0009(0.0005-0.0013) | 0.001(0.0006-0.0013) |
| 10 years | 0.002(0.0014-0.0026) | 0.0033(0.0027-0.004) |
| 15 years | 0.0036(0.0027-0.0045) | 0.0061(0.0051-0.007) |
